# Editorial: Defining Construction: Insights Into the Emergence and Generation of Linguistic Representations

**DOI:** 10.3389/fpsyg.2021.781483

**Published:** 2021-11-03

**Authors:** Matthew T. Carlson, Antonio Fábregas, Michael T. Putnam

**Affiliations:** ^1^Department of Spanish, Italian, and Portuguese, and the Program in Linguistics, Penn State University, State College, PA, United States; ^2^Department of Language and Culture, The Arctic University of Norway – UiT, Tromsø, Norway; ^3^Department of Germanic and Slavic Languages, and the Program in Linguistics, Penn State University, State College, PA, United States; ^4^Centre for Research & Enterprise in Language, University of Greenwich, London, United Kingdom

**Keywords:** constructions and lexicon, generative syntax, emergentism, morphology, competence and performance

## Introduction

A universal goal and challenge in linguistic theorizing is to understand the abstract elements or representations that explain how human know and use language, and how those elements interact with one another. For the sake of exposition, we label such structures as CONSTRUCTIONS without making a commitment to any particular framework or set of assumptions. Some notion of construction seems to transcend across different frameworks (i.e., proof- vs. model-theoretic), across positions regarding the nature of human linguistic competence (i.e., nativist vs. emergentist) and also across different conceptions about performance biases and so-called third factor criteria (Chomsky, 2005).

The contributions to this Frontiers of Psychology Project, *Defining Construction: Insights Into the Emergence and Generation of Linguistic Representations*, address larger-scale questions concerning the emergence and classification of constructions both from specific theoretical points of view and in efforts to build on multiple perspectives toward new and useful synergies, raising new questions and pushing the traditional boundaries of research on linguistic structure. A particularly salient thread that finds its way into each of these contributions is the debate between the existence of language-particular constructions (Goldberg, 2006) and “universal” derivational procedures that act upon other axioms (i.e., features) of linguistic competence (Chomsky, 1977), and each of the contributions handles this thread in its own way.

## Summary of Contributions

In total, this Frontiers in Psychology Project contains nine contributions which address various aspects of current research on the notion of constructions.

Káldi et al. concentrate on focused elements in Hungarian in “*Hungarian structural focus: Accessibility to focused elements and their alternatives in working memory and delayed recognition memory*.” Based on findings of enhanced activation of focused targets in working memory, but greater activation of alternatives after a short delay, they argue that preverbal focus temporarily enhances attention to the target, but that focused structures also broaden the scope of attention to alternatives.

Nicoladis and Sajeev investigate the role of surface-level and abstract representations in the developing grammar of bilingual children in “*Developing abstract representations of passives: Evidence from bilingual children's interpretation of passive constructions*.” Their study confirms that the development of more abstract linguistic representations is closely tied with language usage.

Cannizzaro and Hendriks address the paramount question of whether production can in fact precede comprehension in L1 acquisition in “*Production before comprehension in the emergence and transitive constructions and Dutch child language*.” Using the constraint-based paradigm of Optimality Theory, Cannizzaro and Hendriks model the conflict between constraints on word order and animacy that facilitate the successful acquisition of transitive structures.

Trotzke examines the domain-specific nature of cyclicity in Minimalism in, “*Constructions in Minimalism: A functional perspective on cyclicity*.” Trotzke makes the case that “atomic” items in syntactic derivations can be of arbitrary length. As a result, the opposition between traditional “words” and larger units of constituency “phrases” is arbitrary. Focusing on examples of subextraction, Trotzke suggests the extant evidence supports that performance, rather than syntactic structure, is the primary culprit for ill-formedness.

Jackendoff and Audring present a novel approach to word structure in “*Relational Morphology: A cousin of Construction Grammar*.” In Relational Morphology (RM), which they interpret as a framework closely related to Construction Grammar (CxG), the conceptualization of the traditional lexicon is extended and incorporated into the parallel architecture framework (Jackendoff, 1997). Here they demonstrate how their notion of *schema* enriches CxG's notion of *construction* in a number of important and conceptually-appealing ways.

Endresen and Janda provide a case study of grammatical constructions and how they function in a single language (Russian) in “*Taking Construction Grammar on step further: Families, clusters, and networks of evaluative constructions in Russian*.” Endresen and Janda utilize the *Russian Constructicon*, a multi-word open-access resources shared between The Arctic University of Norway-UiT and the National Research University Higher School of Economics in Moscow.

In his article, “*What are constructions, and what else is out there? An associationist perspective*,” Kapatsinski takes aim at evaluating the validity of bidirectional form-meaning associations in connection with language comprehension and production. Kapatsinski advances arguments in favor of bidirectional form-meaning associations from a Constructionist approach, showing that the complex interplay of both positive and negative form-meaning associations plus paradigmatic mappings provide nuanced insights into the properties of the *bez*-adjective construction in Russian.

Carlson et al. pose timely and important questions concerning the similarities and differences of how generative and usage-based approaches conceptualize the notion of “construction” in, “*How wide the divide? – Theorizing ‘constructions' in generative and usage-based frameworks*.” At the heart of this positional piece, Carlson et al. elucidate areas of commonality across these traditionally divergent approaches, while also pointing out key differences in the way both sets of scholars working within these frameworks interpret the ontology of “constructions.”

Finally, the contributions to this project concludes with Michaelis and Hsiao's contribution entitled, “*Verbing and linguistic innovation*.” In this study Michaelis and Hsiao home in on the process of *conversion*, according to which a lexical item's inflection and combinatory potential change while its internal composition does not. Michaelis and Hsiao take a closer look at denominal verbs in English, revisiting, and in some ways, reappraising the claims associated with Clark and Clark's (1979) seminal paper “When Nouns Surface as Verbs.” These authors argue that “syntacticized” approaches to semantic representation fail to account for the full range of interpretable strategies used by English speakers in created denominal verbs, while at the same time pointing toward context-independent systematicity in this phenomenon.

The breadth of issues, perspectives, and questions that the articles contained in this collection address suggests an emerging, though complex picture, indicating that the problem is far from settled, but that the field will greatly benefit from a more intense cross-theoretical discussion. Findings showing that abstraction increases with usage (Nicoladis and Sajeev), production can precede comprehension in development (Cannizzaro and Hendriks), the explanatory power of negative associations within a construction-based view (Kapatsinski), context-dependent derivational processes (Michaelis and Hsiao), and the need for units of arbitrary size in syntactic derivations and the lexicon (Carlson et al.; Jackendoff and Audring; Trotzke) points toward an origin of abstraction in language-specific representations, often at a fairly large scale, with long-term (permanent?) traces on the associated grammars, i.e., constructions in something like the sense of Goldberg (2006). On the other hand, the role of context-independent processes (Michaelis and Hsiao), and systematicity in how abstraction develops (Cannizzaro and Hendriks) and is used (Carlson et al.; Endresen and Janda; Kaldi et al.; Trotzke), points toward cross-linguistic consistency the kinds of abstraction that operate to different degrees across development and maturity. In our opinion, this collection illustrates how all of this must be incorporated into our understanding of the nature and sources of abstraction in human language, pointing toward rich directions for research in the immediate future, which we hope this collection will encourage.

## Author Contributions

All authors listed have made a substantial, direct and intellectual contribution to the work, and approved it for publication.

## Conflict of Interest

The authors declare that the research was conducted in the absence of any commercial or financial relationships that could be construed as a potential conflict of interest.

## Publisher's Note

All claims expressed in this article are solely those of the authors and do not necessarily represent those of their affiliated organizations, or those of the publisher, the editors and the reviewers. Any product that may be evaluated in this article, or claim that may be made by its manufacturer, is not guaranteed or endorsed by the publisher.
